# Gamer in the scanner: Event-related analysis of fMRI activity during retro videogame play guided by automated annotations of game content

**DOI:** 10.1162/IMAG.a.1256

**Published:** 2026-06-01

**Authors:** Yann Harel, Basile Pinsard, Julie Boyle, Valentina Borghesani, Maximilien Le Clei, Paul-Henri Mignot, Marie St-Laurent, Karim Jerbi, Lune Bellec

**Affiliations:** Department of Psychology, University of Montréal, Montréal, Canada; Centre de Recherche de l’Institut Universitaire de Gériatrie de Montréal (CRIUGM), Montréal, Canada; Swiss National Centre of Competence in Research, University of Geneva, Geneva, Switzerland; IMT Atlantique, Brest, France; Mila – Quebec AI Institute, Montréal, Canada; UNIQUE – Union Neurosciences et Intelligence Artificielle – Québec, Québec, Canada

**Keywords:** naturalistic neuroimaging, video games, automated annotation, fMRI, cognitive neuroscience

## Abstract

In recent years, videogames have gathered interest in cognitive neuroscience for their potential to study cognition in dynamical and naturalistic contexts. Yet, the complexity of game environments often challenges traditional modeling approaches, and current annotation methods—typically manual or based on modified games—remain labor-intensive and limited in scope. Here, we introduce a flexible and scalable framework using the gym-retro Python library to emulate a classic action-platformer, *Shinobi III: Return of the Ninja Master*, and automatically annotate gameplay events directly from the game’s memory states. This setup enables the identification of both player actions (e.g., jumping, hitting) and feedback events (e.g., killing an enemy, being hit), without modifying the game. Four individuals played the videogame for a combined total of 32 h (>7 h each) while undergoing functional magnetic resonance imaging (fMRI). Resulting activation maps revealed distributed engagement of visual, motor, executive, and limbic systems, consistent with the cognitive demands of gameplay. Within-participant reproducibility of brain responses across sessions was robust across event types (r ≈ .25–.55), with some consistency observed even for rarer events like HealthLoss. Between-participant correlations were notably lower, reflecting participant-specific neural signatures. Multivoxel pattern analysis showed that brain responses to different in-game events were highly discriminable, with classification accuracy typically around or above 90%, though occasionally dropping to ~40% for less frequent events. These findings demonstrate that automated emulator-based annotations enable robust, interpretable, and scalable mapping of naturalistic cognitive processes using commercial videogames.

## 1 Introduction

A growing trend is to study cognition in naturalistic settings, employing rich, multifaceted paradigms (Herholz et al., 2022; Nastase et al., 2020) such as passive movie watching (Eickhoff et al., 2020; Jääskeläinen et al., 2021; Vanderwal et al., 2019), free reading (Hsu et al., 2019; Wehbe et al., 2014), simulated driving (Choi et al., 2017; Schweizer et al., 2013) and videogame playing. Such naturalistic stimuli, however, present a complex temporal structure, more challenging to model than classical controlled laboratory tasks (Huettel, 2012; Warbrick et al., 2009). Notably, cognitive events often overlap, nest within one another, and may lack a clear onset and duration. To capture the neural underpinnings of complex behavior in naturalistic conditions using a general linear model (GLM, Poline & Brett, 2012) in functional magnetic resonance imaging (fMRI), one thus needs to model the intricate temporal structure of stimuli with precise and detailed annotations.

Since the foundational work of Hasson et al. (2004), movie watching has emerged as a popular paradigm for naturalistic fMRI experiments. Many forms of movie content annotations have been proposed in the literature, ranging from human raters who identify features of interest manually, such as scenes and characters, to the deployment of machine learning automated tools (Wallenius, 2010). In particular, the Neuroscout platform enables automated movie annotations that capture elements like the presence of faces and buildings, the frequency of words spoken, and other audio-visual features (de la Vega et al., 2022). Movie watching tasks are characterized by a unidirectional flow where the stimulus remains constant irrespective of the participant’s response. In stark contrast, videogames introduce a bidirectional interplay (Matusz et al., 2019) where the environment actively responds and adapts based on the participant’s actions and decisions (Potter et al., 2014). This makes videogames a powerful tool to probe cognitive domains that passive naturalistic tasks cannot access, such as psychomotor learning, decision-making, and problem solving. However, the dynamic nature of videogames heightens the need for annotations that can accurately capture these nuanced interactions, and our work proposes a novel high-throughput framework to generate videogame event annotations and corresponding brain activation maps.

Several studies analyzed fMRI data acquired during complex videogame play. Many of these have relied on human raters to annotate game sequences. Weber and colleagues (Mathiak & Weber, 2006; Weber et al., 2006) have employed frame-by-frame handmade annotations to identify relevant variables during First Person Shooter (FPS) gameplay, such as passive/dead states, preparation/search phases, potentially dangerous encounters, and moments when the player character is under attack. Wang et al. (2018) recruited 70 players to play *League of Legends* in a 3T MRI, and had six experimented raters annotate 15-s segments assessing the quality of in-game maneuvers as either good, average, or poor plays. They then used general linear models (GLMs) and seed-based functional connectivity to uncover neural correlates of gameplay proficiency. Zhang et al. (2021) enlisted two human raters to annotate game events during replays of *Counter-Strike: Source* such as kills, deaths, instances of safe or unsafe exploration, engagements, use of flashbang grenades, and both distant and close engagements. While human videogame annotations can achieve granularity and depth, this approach is prohibitively labor-intensive and cannot easily be scaled to large datasets.

Another approach to capturing in-game events is to incorporate modifications directly into game software to log events and annotate fMRI signal. For example, Lim et al. (2019) studied incidental learning using a modified game platform. Within this environment, players encountered distinctive “aliens”, each associated with a particular sound. These occurrences were marked as events within the game’s presentation software, ensuring that they could be readily tracked and analyzed. Similarly, Kätsyri et al. (2013a, 2013b) used a modified version of the open-source game *BZFlag* (Schoeneman & Riker, *BZFlag*) to log each success or failure. These annotations were then used to compare active and vicarious play, and to study brain responses to human or artificial opponents using GLMs. Such modified game software approaches, while also labor-intensive and restricted to open-source or custom-made games, offer the advantage of tailoring the gaming experience to the specific needs and goals of the research study, ensuring that pertinent events are captured accurately and consistently.

While previous research has explored neural patterns evoked during videogame play, most work employed a tedious and incomplete annotation process, resorting to manual annotations or modified game softwares. Our research introduces a new flexible approach that facilitates the annotation of videogame tasks performed in the scanner by integrating console emulators directly into the neuroimaging setup. This approach is made easily accessible via OpenAI’s *gym-retro* (Nichol et al., 2018), a Python library designed to facilitate the development of artificial agents and accelerate machine learning experiments by offering a unified interface with the most common console emulators. The library thus provides access to a vast catalog of retro videogames (i.e., videogames released on older consoles), and offers versatility in the selection and control of game parameters. For the Courtois project on neuronal modelling (CNeuroMod; Boyle et al., 2020), a large-scale deep phenotyping dataset comprising a diverse set of tasks that includes several naturalistic paradigms, we developed a neuroimaging acquisition setup tailored to record brain activity during retro videogame sessions played on a console emulator. Participants each played over 7 h of *Shinobi III: Return of the Ninja Master* (*Shinobi III: RotNM*; Sega, 1993), a classic action platformer, under naturalistic play conditions while undergoing fMRI. We annotated play content with high timing precision using the game’s memory states, which captured diverse in-game events such as kills, health losses and player actions (button presses). We aimed to demonstrate that this high-throughput annotation framework can be used to generate appropriate events for GLM analyses, and more specifically demonstrate that (1) there are enough repetitions of different event types and, (2) different event types have sufficiently small correlations that it is possible to derive reliable and distinct maps of brain networks using a GLM and these annotations. Specifically, we produced individual brain activation maps linked to different types of automatically annotated events and assessed their quality by comparing these against statistical maps obtained from functional localizer tasks acquired in the same participants. With this approach, we evaluated the consistency of these activations between and within participants using spatial correlations measures. We also assessed the distinctiveness of these spatial neural networks with multivoxel pattern analysis (MVPA) classification. Our results indicate that annotations derived automatically from game emulators can greatly facilitate the identification of meaningful brain activity patterns that reflect various game events.

## 2 Methods

### 2.1 CNeuroMod participants

The CNeuroMod project compiled a series of datasets involving six participants (sub-01 to sub-06) who engaged in various tasks over 6 years (Boyle et al., 2020). Informed written consent was obtained from all participants, and the experimental protocol was approved by the “*Comité d’éthique de la recherche vieillissement-neuroimagerie*” of the CIUSSS du Centre-Sud-de-l’île-de-Montréal (CIUSSS-CSMTL). Participants were right-handed English speakers with normal or corrected-to-normal hearing and vision, aged 31 to 47 years at the start of data collection.

### 2.2 Datasets

This study utilizes two CNeuroMod datasets: *shinobi* and *hcptrt*. Due to the scope and duration of the project, not all participants completed all tasks. For this study, we focused on the subset of participants who completed both the *shinobi* and *hcptrt* protocols. A more detailed description of these datasets can be found in the CNeuroMod technical documentation (CNeuroMod, 2025a).

The *shinobi* dataset explores behavioural and brain correlates of videogame play and includes four participants (sub-01, sub-02, sub-04, and sub-06; two females, two males) who played *Shinobi III: Return of the Ninja Master* (RotNM; Sega, 1993) inside an MRI scanner. While not a central focus of this study, prior videogame experience may have influenced the results. Among the participants, sub-01 had prior experience with *Shinobi III: RotNM*, and three participants (sub-01, sub-02, and sub-04) reported regular videogame play. One participant (sub-06) reported no videogame experience.

The *hcptrt* dataset includes a range of tasks designed by the Human Connectome Project (Barch et al., 2013), repeated multiple times per participant to generate brain maps of various systems. For this study, we used *hcptrt* data from the same four participants who completed the *shinobi* acquisitions. The dataset encompasses seven distinct tasks, each administered up to 15 times per participant, resulting in approximately 10 h of fMRI data per participant (Rastegarnia et al., 2023). Here, *hcptrt* serves as a benchmark for assessing the consistency of brain maps derived from established functional localizer paradigms and evaluating whether brain maps generated from videogame data align with those from conventional localizers.

### 2.3 Behavioral acquisitions

#### 2.3.1 Videogame interface

The videogame was played on a console emulator using OpenAI’s *gym-retro* library (Nichol et al., 2018), a Python-based platform supporting emulators for over 10 retro consoles and thousands of games. Built on *gym* (Brockman et al., 2016), a library designed for reinforcement learning, *gym-retro* integrates console emulators via the Libretro API (Libretro, n.d.). This setup allows both artificial and human agents to play retro games from saved states and provides access to the game’s rapid-access memory (RAM) through a Python API.

In this experiment, *Shinobi III: Return of the Ninja Master* (RotNM) was played and recorded at a 60 Hz framerate. Because the game is fully deterministic (identical inputs produce identical results), only player inputs (button presses) were recorded, enabling the reconstruction of exact replays of gameplay sequences. These replays facilitated a posteriori RAM mining, mapping emulator memory addresses and retrieving value changes in identified variables.

#### 2.3.2 Videogame task

*Shinobi III: RotNM* (Sega, 1993) is a classic action platformer released on the Sega Genesis console. The platformers genre was selected by the CNeuroMod because gym-retro was developed for a major reinforcement competition centered on this genre (OpenAI, 2018). This game was further selected for its compatibility with *gym-retro* and its commercial success, ensuring engaging gameplay. As in other platformers, players navigate a linear two-dimensional environment filled with enemies and obstacles to reach a destination. For this study, three levels or portions of levels (1-0, 4-1, and 5-0; abbreviated as 1, 4, and 5) were selected for their gameplay similarity, despite varying difficulty.

In all selected levels, players began on the far left and progressed to the far right using seven primary actions: move left, move right, crouch (move down), hang (move up), jump, attack, and power up. By combining buttons or executing timed sequences, players could perform additional actions such as dashing (forward, forward), wall-jumping (JUMP + LEFT/RIGHT against a wall), low-kicking (DOWN + HIT near an enemy), and jump-kicking (JUMP + DOWN + HIT near an enemy). Levels 1 and 5 concluded with a boss fight against a stronger enemy.

**Fig. 1.**
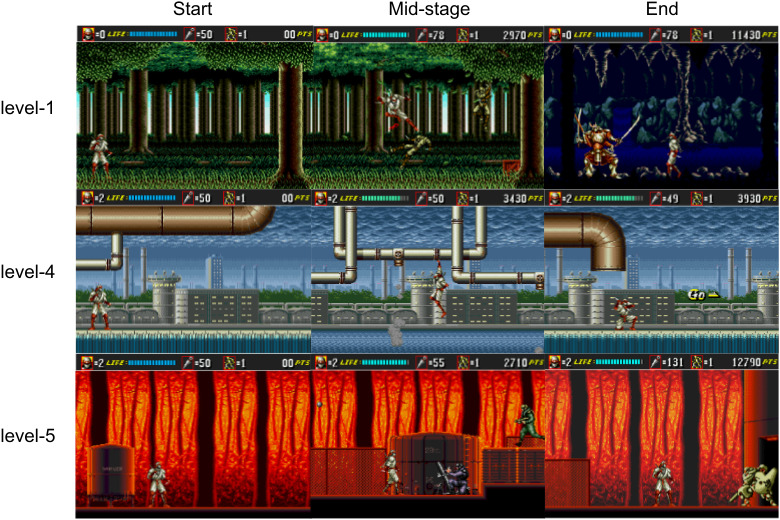
Examples of game frames from the three selected levels. Shown are the first frame of each level (“Start,” left), an arbitrarily selected mid-level frame (“Mid-stage,” middle), and a final frame (“End,” right) depicting either a boss fight (levels 1 and 5) or the level’s exit (level 4).

Each attempt at completing a level, referred to as a “repetition,” began with 16 health points and 50 projectiles. Players needed to complete the level, including the boss fight, without dying. Bonuses scattered throughout the levels allowed players to restore health or replenish projectiles. A selection of game frames is shown in Figure 1.

### 2.4 Neuroimaging acquisitions

#### 2.4.1 MRI sequences

MRI data were collected using a 3T Siemens PrismaFit scanner with a 64-channel head-neck coil at the functional neuroimaging unit (UNF) of the Centre de Recherche de l’Institut Universitaire de Gériatrie de Montréal (CRIUGM) in Montréal. Additional acquisition details are available in the CNeuroMod technical documentation (CNeuroMod, 2025c).

Anatomical data were acquired during separate dedicated sessions using a T1-weighted MPRAGE 3D sagittal sequence (duration 6:38 min, TR = 2.4 s, TE = 2.2 ms, flip angle = 8 deg, voxel size = 0.8 mm isotropic, R = 2 acceleration). These scans demonstrated high structural stability of individual brain anatomy throughout the CNeuroMod project (Boudreau et al., 2025).

Functional data were acquired using an accelerated simultaneous multi-slice gradient echo-planar imaging sequence from the Human Connectome Project (slice acceleration factor = 4, TR = 1.49 s, TE = 37 ms, flip angle = 52°, 2 mm isotropic spatial resolution, 60 slices, 96 x 96 acquisition matrix; Setsompop et al., 2012).

#### 2.4.2 MRI acquisition set-up

Visual stimuli were projected using an Epson Powerlite L615U onto a blank screen behind the MRI bore, viewed through a head coil-mounted mirror. Audio was delivered via S15 Sensimetrics headphone inserts. To minimize motion artifacts and improve fMRI signal quality, the CNeuroMod project employed individualized polystyrene head cases (CaseForge; Lynch et al., 2021). Physiological data were recorded concurrently with neuroimaging but are not within the scope of this study.

#### 2.4.3 MRI-compatible videogame interface

During neuroimaging sessions, the game was presented using a Python software stack (CNeuroMod, 2025e) that employed PsychoPy (Peirce et al., 2019) to synchronize task onset with scanner trigger pulses, initiate the gym-retro library with predefined save states for specific levels, and record player inputs (button presses). The software also produced real-time outputs (sound and video frames) for participants and detected conditions for ending level repetitions (player death or level completion).

Participants used a custom MRI-compatible videogame controller, designed with 3D-printed plastic and fiber optics, connected via USB to the stimulation computer. Developed by our team, this controller allowed high performance gameplay while effectively minimizing acquisition artefacts caused by scanner interference. To promote reproducible science, the design plans were publicly shared (Harel et al., 2023).

The controller replicated the layout of a SuperNES™ controller (Inoue & Ashida, 1993), featuring two sets of four buttons accessible via the thumbs. For this task, the left buttons controlled character motion (RIGHT, LEFT, UP, DOWN), while the right buttons controlled actions (JUMP, HIT), mirroring the original console’s button layout.

#### 2.4.4 MRI preprocessing

Anatomical data were preprocessed using the sMRIPrep pipeline, with T1w and T2w scans from the first two anatomical sessions of all participants as input. These scans were coregistered and averaged to enhance structural stability. Functional MRI data were preprocessed using fMRIPrep 20.2 LTS (Esteban et al., 2019), built on Nipype 1.6.1. Confounds time series for subsequent analyses were derived from the fMRIPrep pipeline output.

We employed the “simple” denoising strategy, including high-pass filtering, regression of average signal within white matter and ventricles, and motion parameters, following the recommendations of Wang et al. (2024) for datasets with low motion levels. This approach preserved temporal continuity in the denoised time series. Detailed preprocessing steps are available on the CNeuroMod website (CNeuroMod, 2025b). For subsequent analysis, the confounds time series were added to the design matrix using the following Nilearn command:

confounds = nilearn.interfaces.fmriprep.load_confounds(

fmri_fname,

strategy=('motion', 'high_pass', 'wm_csf'),

motion='full',

wm_csf='basic'

)

#### 2.4.5 The shinobi dataset

##### 2.4.5.1 At-home training

**Appendix Fig. A1.**
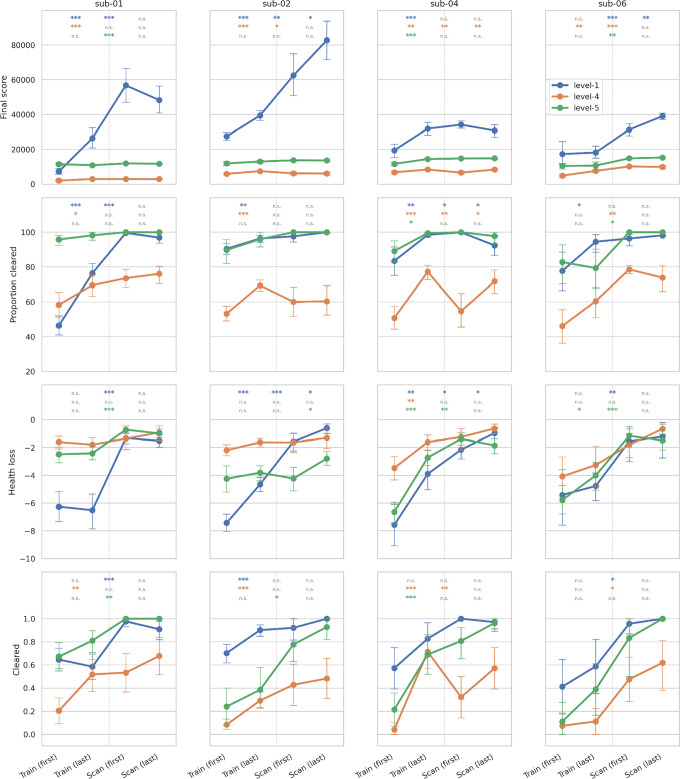
Skill acquisition across training and scanning phases. For each level and participant, training and scan sessions were each split at their midpoint (in order of repetitions) into a first and second half, and the three consecutive half-pairs were compared: within training, from training to scanning (transfer), and within scanning. Significance was assessed with two-sided Welch’s t-tests, FDR-corrected using the Benjamini-Hochberg procedure: * *q* < .05; ** *q* < .01; *** *q* < .001.

All participants were given extensive time to practice during the weeks or months preceding neuroimaging. By the start of the experiments, all participants were familiar with the game, as indicated by clear progress on performance metrics. Corresponding behavioral practice data are publicly available as the *shinobi_training* dataset (CNeuroMod, 2025f). A table describing breaking down the amount of training time per participant and per level is presented in Appendix Table A1, and a comparison of the performance between at-home training and in-scanner sessions is presented in Appendix Figure A1.

##### 2.4.5.2 In-scanner play sessions

Participants played *Shinobi III: RotNM* to the best of their ability while undergoing MRI scanning. The shinobi dataset also served as a pilot for the gym-retro interface in preparation of future videogames acquisitions via our platform. During each fMRI run, participants sequentially played the three selected levels (1, 4, and 5) for a minimum of 10 consecutive minutes. Runs were manually stopped at the end of the ongoing repetition after 10 min, regardless of the level reached. If a participant died or cleared a level, they immediately proceeded to the next one. Each fMRI session included 4–5 runs, with total playtime approaching 7 h for sub-06 and 8 h for all other participants (see Table 1; a more comprehensive table of descriptive metrics is provided in Supplementary Materials as SM1). Each participant completed 12 sessions, except sub-04, who completed 11. The behavioural and neuroimaging data are publicly available as the *shinobi* dataset (CNeuroMod, 2025d). Session 009 of sub-06 was ignored in subsequent analysis because of a software issue that prevented the recording of behavioral data.

**Table 1.** Descriptive table of the *shinobi* dataset.

		sub-01	sub-02	sub-04	sub-06	Total
level-1	N	87	77	67	46	277
	cleared	82	74	66	45	267
	duration (h:m:s)	03:51:53	03:31:10	03:17:55	03:29:24	14:10:22
level-4	N	61	57	56	42	216
	cleared	37	26	25	23	111
	duration (h:m:s)	01:54:00	02:09:48	02:06:00	02:44:22	08:54:11
level-5	N	53	55	53	12	173
	cleared	53	47	47	11	158
	duration (h:m:s)	02:05:45	02:37:35	02:39:48	00:45:31	08:08:38
Total	sessions	12	12	11	11	46
	runs	53	55	55	47	210
	N	201	189	176	100	666
	cleared	172	147	138	79	536
	duration (h:m:s)	07:51:38	08:18:33	08:03:42	06:59:18	31:13:11

#### 2.4.6 Quality control

We extracted the following QC measures from fMRIprep (Esteban et al., 2019) and the MRIQC BIDS app (Provins et al., 2023):

- Framewise displacement (FD) summary measures: the mean and the max value of each run. Max value will show the worst instantaneous motion in each run.
- Motion extent or excursion, as the max difference between max and min values of each of the 6 motion parameters.
- Temporal signal to noise ratio (TSNR) averaged in the brain mask, as extracted by MRIQC.

Crowdsourced MRIQC measures were pulled from the MRIQC API, filtering for approximately matching acquisition parameters in terms of magnetic field strength, TR, voxel size, and multiband acceleration factor, and with minimum duration of 100 TRs. A figure showing the run-median FD values is presented in SM3 Supplementary Figure S1.

### 2.5 Annotation of task events

#### 2.5.1 Memory (RAM) mining

**Fig. 2.**
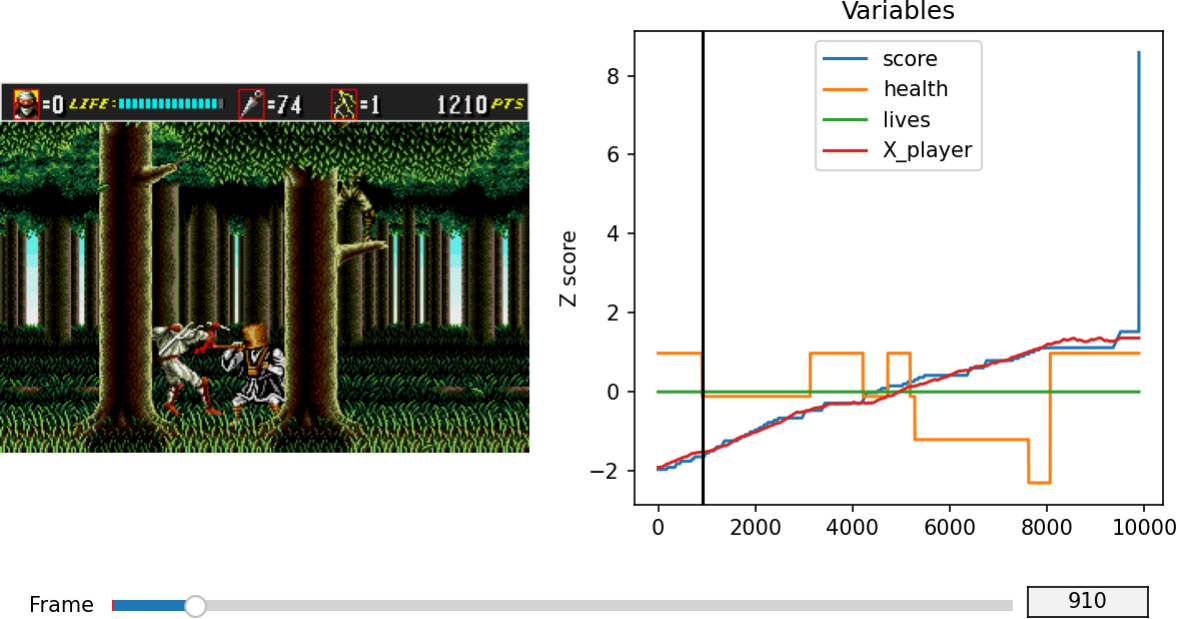
Exploration of variables in the memory during replay. Capture of a selected game frame (910; left), and time course (in frames; right) for normalized variables *score* (blue), *health* (yellow), *lives* (green) and *player horizontal position* (“X_player”, red) for one level repetition. At the selected frame (black vertical line), the player has been hit by an enemy and the health variable (yellow) shows a concomitant decrement.

The videogame console’s memory stores temporary variables that encode the current game state. The *gym-retro* library includes an integration software for researchers to explore and map memory addresses in order to extract meaningful game variables. This software allows users to play the game, to log performance and progress metrics and to track value changes in the memory. Replay files are exported in a *gym-retro*-specific format called bk2. Replay bk2 files encoded all button presses and could be used with *gym-retro* to regenerate gameplay videos and produce a complete mapping of memory states for each game step (see Fig. 2). The process of mapping game variables to memory addresses is a feat of reverse engineering. The *gym-retro* library provides a graphical interface that allows the reading, lookup and modification of RAM values as the game is running within the emulator. Through monitoring memory changes as the game unfolds while deliberately manipulating the content of target variables (e.g., dying on purpose, moving left or right etc.), we deduced the location of variables encoding the *score*, *health*, *lives* and *player horizontal position*.

#### 2.5.2 Game-related events

We first produced player **action events** that marked the timing of button presses and releases. While we recorded all possible actions, current analyses ignore the *POWERUP*, *START*, *SELECT* and move *UP* actions that were either too sparse or didn’t meaningfully impact the game. Instead, analyses considered the (move) *RIGHT*, *LEFT* and *DOWN* actions, as well as the *JUMP* and *HIT* actions. We then generated two types of game **annotation events** from memory variables, designed to parallel rewarding and punishing events in reinforcement learning. This second type of events does not map one-to-one to button-presses, and constitutes the key innovation of our memory mining approach, expanding event-based analysis to game outcomes without relying on manual annotations. We thus annotated the following events:

- *Kill* corresponds to moments when the player kills an enemy, and carries positive affective valence. *Kill* events are detected with a sudden *score* increase of 200, 300, 400 or 500 points, depending on enemy type.
- *HealthLoss* corresponds to moments when the player loses health when hit by an enemy or trap, which carries a negative affective valence. This event is indicated by a negative change of the *health* variable.

#### 2.5.3 Sensory and motor features

Psychophysical properties of the audiovisual stimulus were computed from the game replay files and added to the set of modeled annotations. *Luminance* was computed as the mean grayscale pixel intensity of each video frame. *Optical flow* was estimated between consecutive frames using the Farneback dense optical flow algorithm (OpenCV; pyr_scale = 0.5, levels = 3, winsize = 15), and summarized as the mean flow magnitude per frame. *Audio envelope* was computed as the root-mean-square (RMS) of the audio signal within each video frame window. These three features, originally sampled at the video frame rate (60 Hz), were downsampled to match the fMRI acquisition rate by averaging within TR-length bins (1.49 s). *Button press* count was constructed by counting the total number of controller button presses (all actions, including those not modeled as individual event regressors) occurring within each TR, to capture the overall density of motor actions. All four continuous regressors were then convolved with the SPM canonical HRF before being entered into the GLM as task regressors. Like game-event annotations, these low-level features were fitted one at a time in separate single-regressor GLMs.

#### 2.5.4 Data structure for events

Task-related events were structured according to guidelines defined by the BIDS (Brain Imaging Data Structure) standard (Gorgolewski et al., 2016). For each fMRI run, an *event* file was generated in .tsv format, named *_desc-annotated_events.tsv. Rows represented different types of events, while columns described features such as occurrence type, onset time, and duration. Specifically, **repetition events** marked the timing of each game level (repetition) within an fMRI run. These events included additional columns for the level played and the path to a corresponding bk2 file. **Annotation events** included Kill and HealthLoss, which were given an arbitrary duration of 0 s. **Action events** included all the button presses, with durations calculated as the interval between button press and release.

### 2.6 Statistical analyses

#### 2.6.1 Regressors

**Fig. 3.**
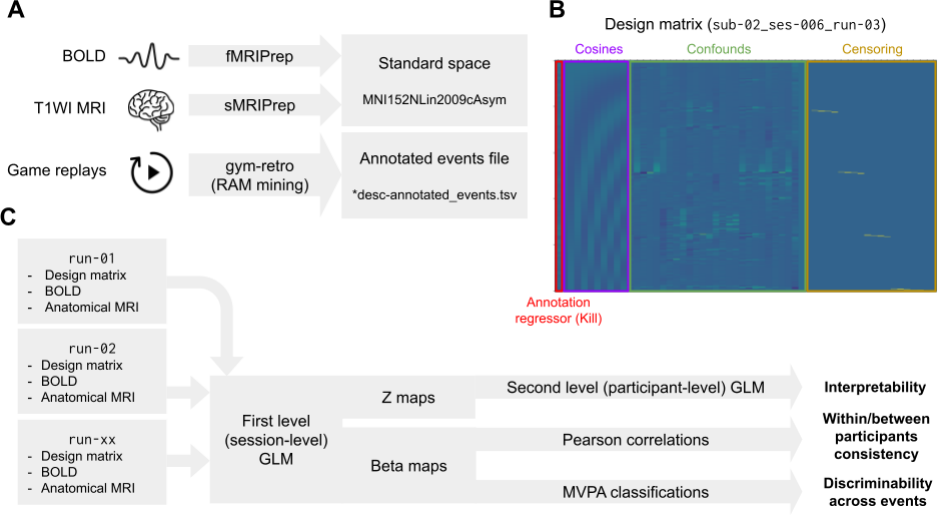
Graphical summary of the analysis pipeline applied to MRI and behavioral data. (A) Describes the preprocessing steps. (B) Shows an example of a GLM design matrix built by combining the preprocessed data. (C) Describes the analysis pipeline used to assess the quality of brain activation maps derived from game events.

A general linear model (GLM) (Poline & Brett, 2012) was used to model the effects of seven game-related events on the BOLD signal after full preprocessing (see Fig. 3A for an overview). Seven game events were considered: RIGHT, LEFT, DOWN, HIT and JUMP player actions, along with Kill and HealthLoss annotations. All analyses were performed using Nilearn (Abraham et al., 2014).

Confounds exported by fMRIPrep were included as additional columns to the design matrix. To further account for potential motion and task-unrelated processes during resting periods between gameplay repetitions, the frames corresponding with these periods were censored using an additional set of one-hot regressors to the design matrix.

#### 2.6.2 First-level (session) general linear model

We tested the main effect of each annotation regressor separately, by fitting a series of single-regressor GLMs. For each model, the annotation time series was convolved with the SPM canonical HRF, and fitted to the smoothed BOLD data (FWHM = 5 mm). fMRIPrep confound regressors (6 motion parameters, white matter, CSF, and global signal) along with high-pass filtering regressors were included in all models. See Figure 3B for an example of a full design matrix for one run. Session-level (first-level) models combined runs from the same session using a fixed-effects approach with an AR(1) noise model. F-tests were used to assess the main effect of each regressor, yielding z-score maps and effect estimates (beta values). The resulting z-maps were corrected for multiple comparisons using voxel-wise FDR (q < 0.05), with an additional minimum cluster extent of 10 voxels.

#### 2.6.3 Second-level (participant) general linear model

Second-level models were computed for each participant by fitting an intercept-only design (equivalent to a one-sample F-test) over all of their uncorrected session-level z-score maps, using nilearn’s SecondLevelModel with no additional smoothing. This resulted in one participant-level map of standardized activations per annotation. The participant-level z-score maps were corrected for multiple comparisons using voxel-wise FDR (q < 0.05) with a minimum cluster extent of 10 voxels. The corrected maps were then clustered (voxel threshold z(F) > 3, minimum cluster size > 5 voxels) using atlasreader 0.3.2 (Notter et al., 2019) for AAL projection, in order to generate a summary of the AAL regions where significant clusters were found (see SM3 Supplementary Table S1).

#### 2.6.4 Brain activation maps derived from HCP test-retest dataset

To assess the quality of brain activation maps derived from game events, we compared these with activation maps obtained from tasks targeting diverse cognitive domains. Each task engaged a distinct subset of cognitive processes, structured through experimental conditions designed to facilitate precise identification of task-related neural activity. Brain activation maps for each task were generated using a GLM at the run level, modeling the main effect of single experimental conditions. These tasks and contrasts are described in detail elsewhere (Barch et al., 2013; Pinho et al., 2018; Rastegarnia et al., 2023) and summarized below:

**Gambling:** participants guessed whether a hidden number between 1 and 9 was above or below 5 to earn a symbolic monetary reward, simulating reward-based decision-making. Stimuli were biased toward either reward or punishment, with trials presented in blocks. Participants were unaware of each block’s nature (reward or punishment). Effects: reward, punishment.

**Motor:** participants performed specific movements (left/right finger tapping, left/right toe squeeze, tongue movement) in response to visual cues to identify motor control regions. Effects: left_hand, right_hand, left_foot, right_foot, tongue, cue.

**Language Processing:** this task assessed language and numerical reasoning. Participants either listened to an auditory story or solved math problems (addition and subtraction of varying lengths). Each trial was followed by a forced-choice question with two alternatives. Effects: story, math.

**Social Cognition:** participants watched video clips of objects (squares, circles, triangles) that either interacted in a socially meaningful way (mental condition) or moved randomly (random condition). They were tasked with judging social intent. Effects: mental, random.

**Relational Processing:** this task assessed relational reasoning. In the relational condition, participants determined whether a pair of objects differed along the same dimension (shape or texture) as a reference pair. In the match condition, they judged whether an object matched one of two options based on a specified dimension. Effects: relational, match.

**Emotion Processing:** participants identified which of two faces (angry or fearful) matched a third face, or which of two shapes matched a reference shape. They were not explicitly instructed that faces conveyed specific emotions. Effects: face, shape.

#### 2.6.5 Beta maps correlations

To assess the inter-session consistency of beta maps derived from game-based annotations, we computed Pearson correlations between voxel-wise beta values for each pair of session-level brain maps associated with the same annotation. We opted for Pearson correlation between whole-brain beta maps as a multivariate measure of spatial pattern similarity, rather than voxel-wise reliability metrics such as ICC. ICC-based approaches decompose variance into within- and between-participant components, requiring a sufficient sample size to robustly estimate between-participant variance, a condition not met in our four-participant dataset. Moreover, voxel-wise reliability metrics tend to underestimate the consistency of distributed activation patterns, even for well-established paradigms (Elliott et al., 2020). In addition, we calculated inter-annotation correlations by measuring the relationship between brain maps from different annotations. This analysis was conducted separately for within-participant correlations (pairs of maps from the same participant) and between-participant correlations (pairs of maps from different participants).

As a reference for reliability estimates, we performed the same analysis on run-level beta maps from the *hcptrt* dataset, which includes diverse event annotations representing motor and cognitive processes. Here, they serve as a benchmark for typical fMRI activation reliability rather than a direct comparison of experimental design principles.

The *shinobi* and *hcptrt* datasets indeed differ fundamentally in design. *Shinobi* activation maps are derived from entire fMRI sessions, as ecological tasks such as videogames naturally allow for extended individual sampling. This prolonged sampling is necessary for certain videogame annotations, such as *HealthLoss*, which occur infrequently in some participants. In contrast, *hcptrt* activation maps are generated from single fMRI runs, with highly controlled designs specifically optimized for that short duration.

#### 2.6.6 MVPA classification

To determine whether each videogame contrast captured unique spatial patterns of brain activity, we conducted an MVPA-based brain decoding experiment using Nilearn (Abraham et al., 2014). For each participant, a linear support vector machine (LinearSVC; scikit-learn, version 1.5.2) was trained on z-scored F maps derived from all session-wise activation maps from *shinobi* (11 regressors, 12–14 sessions per participant), and all run-level activation maps from *hcptrt* (16 regressors, 14–15 runs per participant). In total, the MVPA classified *N* = 27 contrast labels from 345–395 brain maps, independently for each participant. The classifier used default parameters: L2 penalty, squared hinge loss, one-vs-rest multiclass strategy, and regularization strength C = 1.0.

The model was evaluated using a leave-one-session-out cross-validation procedure, training on all sessions (for *shinobi*) and all runs (for *hcptrt*) except one, and testing on the remaining session or run. As part of the cross-validation, feature selection was performed at the voxel level using an ANOVA-based screening method, where voxels were ranked independently, and the top 20% were retained to train the SVM.

Model performance was assessed using a confusion matrix for all 27 classes, with statistical significance determined by comparison against a dummy classifier.

## 3 Results

### 3.1 Game events are frequent and long enough for fMRI analysis

**Fig. 4.**
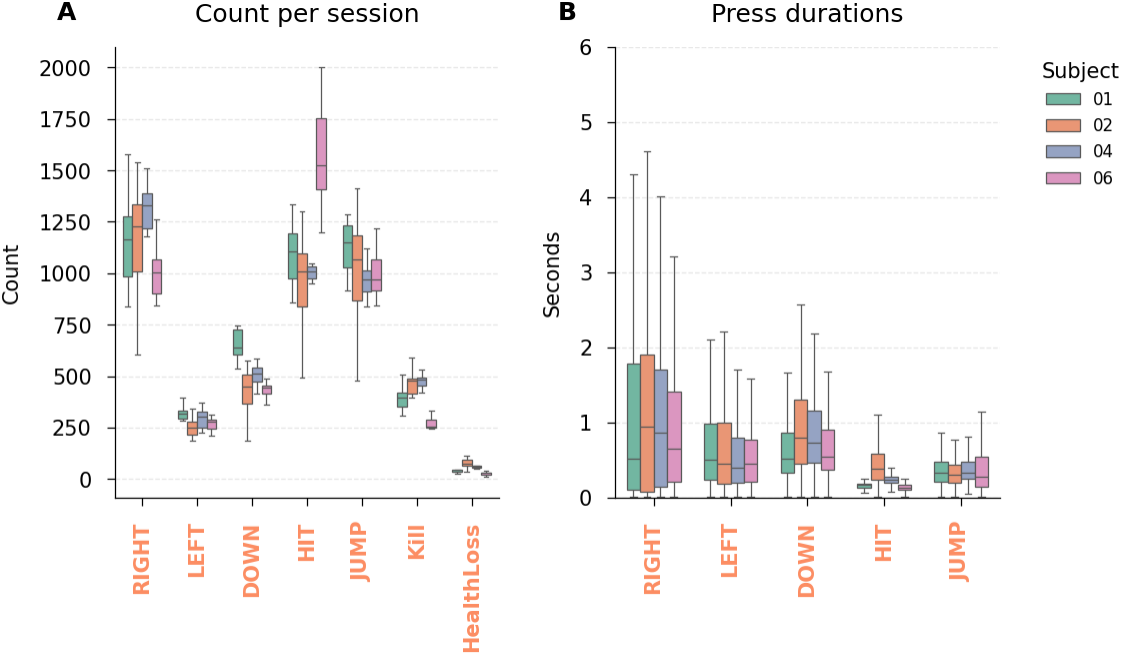
Descriptive statistics of game events. (A) The events count per session. All events except HealthLoss occurred more than 200 times per session, making them appropriate for GLM analysis. (B) The distributions of button press durations. Movement-related button presses (*RIGHT*, *LEFT*, *DOWN*) were sustained for longer durations than *JUMP* and *HIT* button presses, for which duration didn’t meaningfully impact gameplay.

The most common actions were *HIT* (mean = 1,166, range: 491–2,000 occurrences per session), moving *RIGHT* (mean = 1,164, range: 604–1,580), and *JUMP* (mean = 1,041, range: 480–1,595), as these were essential for progressing through the game. *Kill* events were also relatively frequent (mean = 400, range: 234–589), whereas *HealthLoss* events were considerably rarer (mean = 51, range: 9–113) (Fig. 4A).

Keypress durations varied across actions (Fig. 4B). The *RIGHT* button was generally held down for longer durations (mean = 1.27 s, IQR: 0.12–1.73 s), while *LEFT* was used in shorter bursts, primarily for position adjustments and boss fights (mean = 0.67 s, IQR: 0.22–0.88 s). *DOWN* keypresses had intermediate durations (mean = 0.82 s, IQR: 0.38–1.03 s). *HIT*, being a discrete action unaffected by keypress duration, had consistently short occurrences (mean = 0.24 s, IQR: 0.13–0.25 s). *JUMP* duration influenced game behavior, as longer presses resulted in higher jumps, but the mean duration remained relatively short (mean = 0.37 s, IQR: 0.20–0.48 s). Overall, the frequency (~250–1,200 per session, except for *HealthLoss*) and duration (typically several hundred milliseconds) of game events were well-suited for event-related fMRI analyses.

### 3.2 GLM regressors had low to moderate correlations for most game events

In-game actions and events were interdependent. For instance, in *Shinobi III: RotNM*, killing an enemy required executing the *HIT* action. Therefore, a correlation between the *HIT* and *Kill* annotations was expected. More complex dependencies also emerged. For instance, a complex action is the fly kick, which is executed by first pressing *JUMP*, followed by simultaneous pressing *DOWN* + *HIT*.

**Fig. 5.**
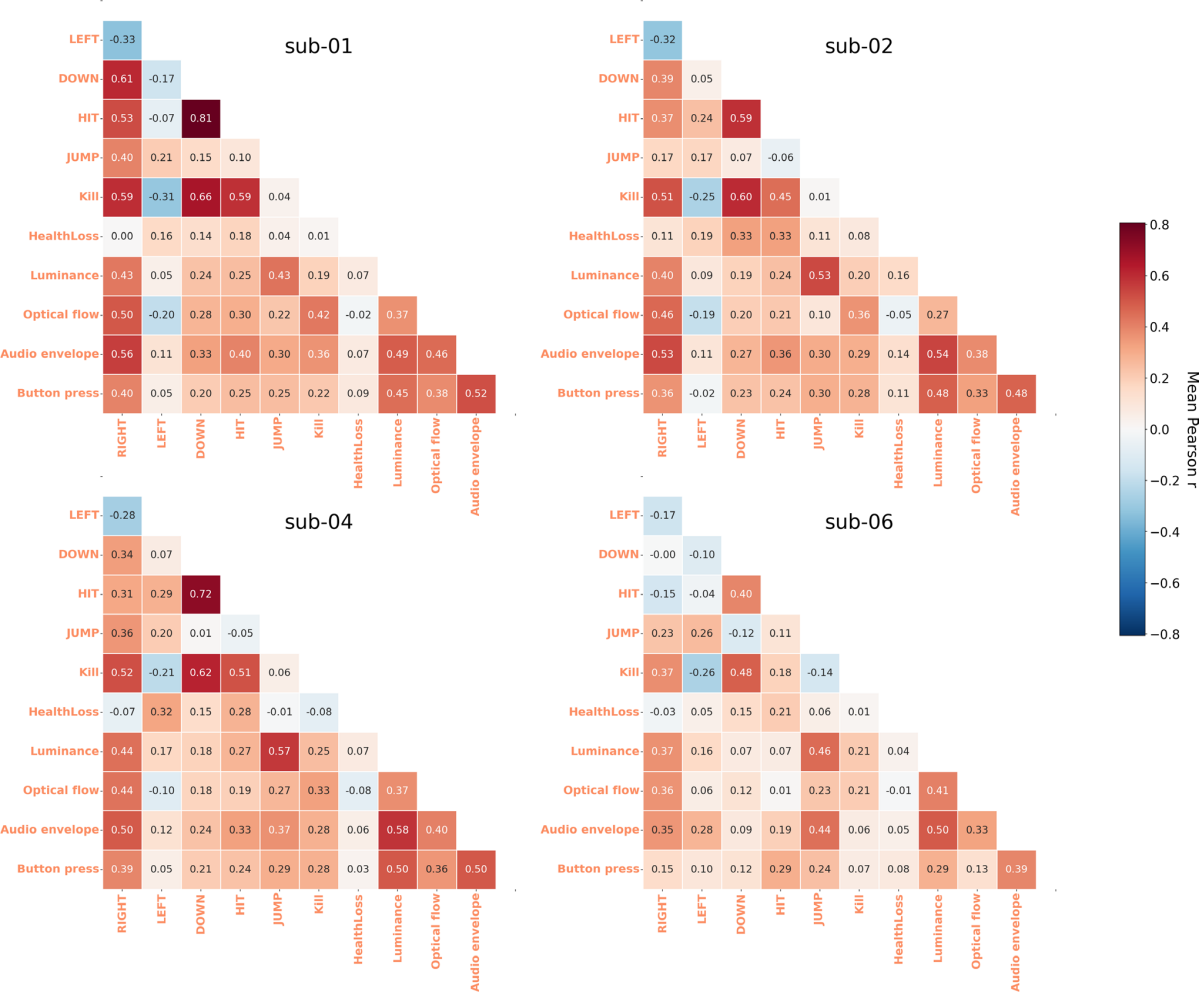
Average Pearson correlation between event regressors. Pearson correlations were computed for each pair of regressors on every run and then averaged per participant to produce correlation matrices. Dependence between game events ranges from very high to non-existent.

To quantify these co-occurrences, regressors were generated for each annotation by convolving a boxcar function with the SPM HRF. Four low-level regressors were additionally included to capture stimulus-driven variance in the BOLD signal unrelated to discrete game events. Pearson correlations were then computed for each pair of regressors on every run and averaged across runs per participant to produce participant-specific correlation matrices (Fig. 5).

As anticipated, *HIT* and *Kill* were moderately correlated (0.18 < r < 0.59), with Kills consistently preceded by *HIT*. *DOWN* also correlated with both *HIT* (0.40 < r < 0.81) and *Kill* (0.48 < r < 0.66). Many pairs of annotations, such as *JUMP* and *HIT* for sub-04, showed low levels of correlations (r < 0.11 across all participants).

Correlations between game annotations and low-level regressors were generally low to moderate. The strongest coupling emerged between *JUMP* and *luminance* (0.43 < r < 0.57), consistent with the abrupt visual changes accompanying vertical movement. *RIGHT* also correlated moderately with audio envelope (0.35 < r < 0.56) and *optical flow* (0.36 < r < 0.50), reflecting the sustained audiovisual dynamics of horizontal movement. Audio envelope showed moderate correlations with most action-related annotations (mean r ranging 0.08–0.49), while *HIT* and *Kill* showed low-to-moderate correlations with all four low-level regressors (r < 0.43). *HealthLoss* was largely decoupled from all low-level features (r < 0.16 in all cases). Among the four low-level regressors themselves, moderate inter-correlations were observed (0.27 < r < 0.58), the strongest being *luminance*–*audio envelope* (0.49 < r < 0.58) and *audio envelope*–*button press count* (0.39 < r < 0.52), reflecting their shared sensitivity to overall gameplay intensity.

Overall, these results indicate that while fMRI regressors derived from game events exhibited some degree of correlation, they captured distinct aspects of gameplay. Given these collinearities, we analysed main effects for each annotation in separate regression models, an approach consistent with the recommendations of Mumford et al. (2015). Our goal was to assess the reliability of brain–behavior associations in videogame play rather than isolate the contributions of individual event types.

### 3.3 Brain activations related to game events reflect sensorimotor system involvement

**Fig. 6.**
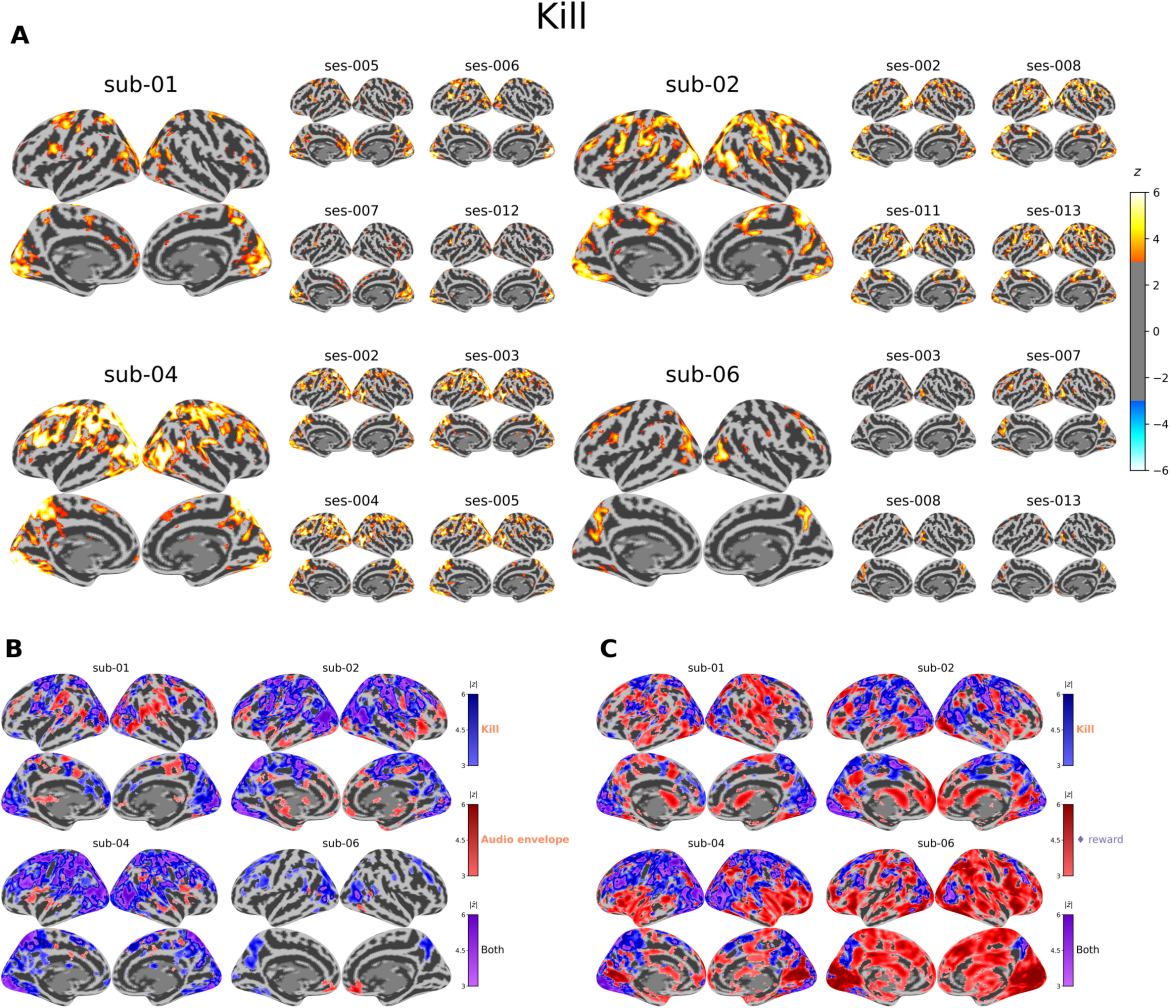
Activation maps obtained with *Kill* (i.e., “killing an enemy”) events. (A) Each panel shows the participant-level maps (left side) and 4 session-level maps (right side) with the highest significant voxel count in the whole brain for each participant. The values shown are z-scores derived from the F-tests, thresholded at z > 3. (B) The conjunction of participant-level z-scores of the *Kill* and *Audio envelope* conditions. Voxels that were activated (3<|z|<6) in the *Kill* condition only are shown in blue (lighter to darker), and voxels activated in the *Audio envelope* condition only are shown in red. Voxels that were activated above threshold (|z|>3) in both conditions are shown in purple, with the average of the two z-scores shown from lighter to darker color. (C) Same as (B) but with the *reward* condition of *hcptrt* instead.

Z-scored statistical maps from second-level models are presented for each participant in relation to the *Kill* annotation (Fig. 6A). A table summary of activated regions using the AAL template in SM3 Supplementary Table S1, and between-participant conjunction maps for all participants is provided in SM3 Supplementary Figure S2. All the generated maps are made available in Supplementary Materials (SM2). The majority of annotations elicited strong bilateral activation in regions associated with visual (superior and middle occipital gyri, cuneus) and sensorimotor (precentral and postcentral gyri, superior parietal gyrus) processing, along with multimodal areas (middle temporal gyrus, precuneus).

Beyond these shared activations, some event-specific patterns emerged. *HIT* and *JUMP* actions were linked to motor regions, including the cerebellum and supplementary motor area, which were not as prominently engaged in the *Kill* and *HealthLoss* conditions. While *Kill* and *HealthLoss* annotations primarily activated visual and sensorimotor networks, they also engaged executive (e.g., superior frontal gyrus) and limbic (e.g., superior and middle temporal cortex) systems. *Kill* events, in particular, elicited activations in ventral (fusiform gyrus) and occipital (calcarine sulcus) visual cortices. Notably, in sub-01, clusters of activation were observed in the left frontal pole and anterior cingulate cortex in response to *HealthLoss* events.

To assess the specificity of the *Kill*-related activation pattern with respect to both low-level stimulus properties and reward processing, Figure 6B and C display three-color surface overlays for each participant. The *Kill* vs. *Audio envelope* comparison (Fig. 6B) reveals that the large majority of cortical activation was shared with the *Audio envelope* (purple), spanning occipital, temporal, and parietal cortices, suggesting that much of the broad visual and multimodal response to *Kill* events was confounded with the low-level sensory signal. *Kill*-unique activations (blue) were more broad and extended to sensorimotor and frontal regions. By contrast, in the *Kill* vs. *reward* comparison (Fig. 6C), *reward*-unique activations (red) spanned nearly the entire cortex across all participants, vastly exceeding the spatial extent of *Kill*-unique activations (blue) which were more localized but included more parietal cortices.

Overall, these findings demonstrate that event-related analyses leveraging automated gameplay annotations yield interpretable activations within visual, sensorimotor, and executive networks.

### 3.4 Within- and between-participant variability of brain maps compared to classical functional localizer tasks

**Fig. 7.**
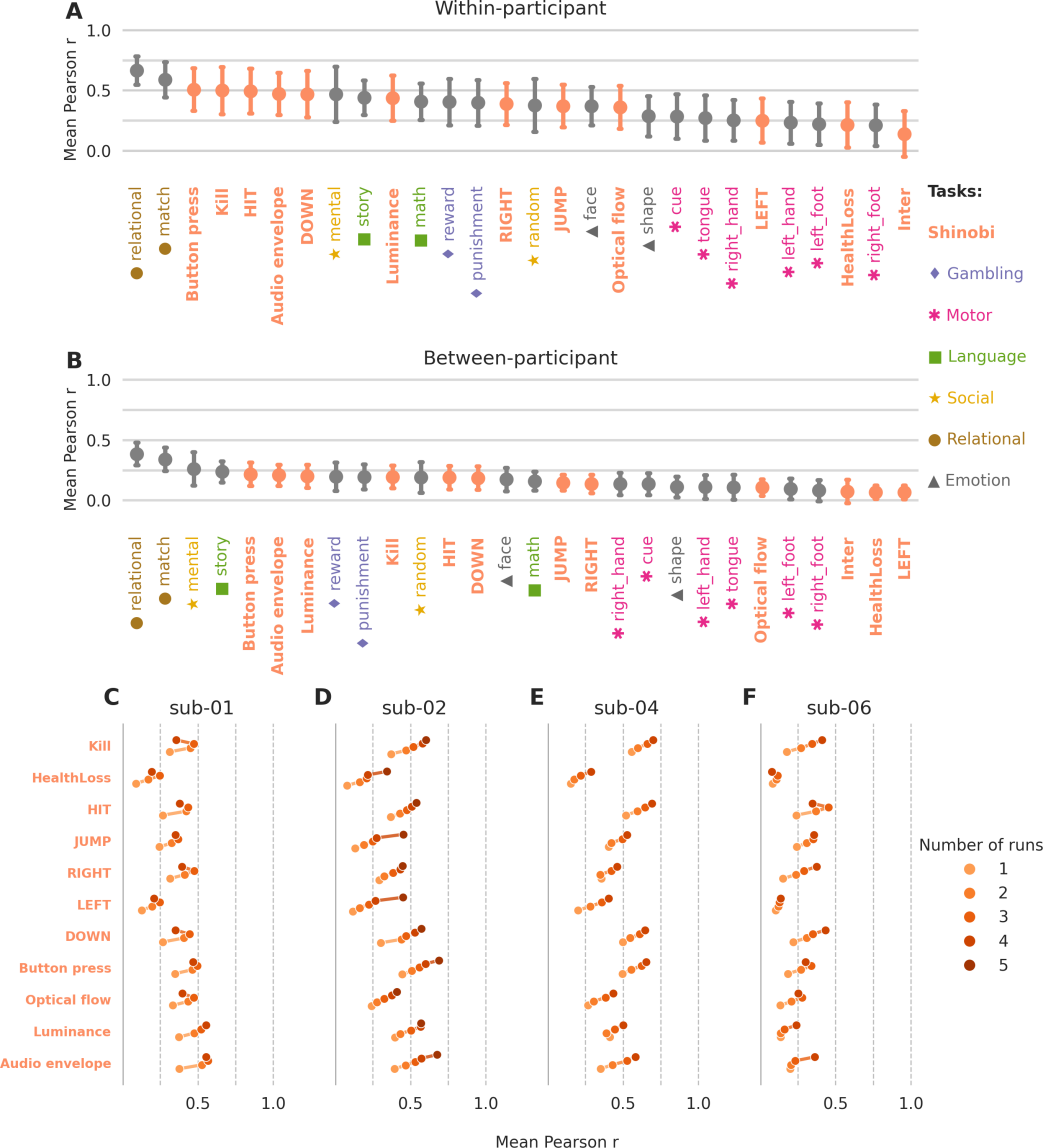
Test-retest consistency of brain activation patterns. The figure presents pairwise Pearson correlations (r) between beta maps derived from single fMRI sessions or runs. Points represent the average correlation between pairs of beta maps for each annotation, computed separately for (A) within-participants and (B) between-participants. Error bars indicate one standard deviation above and below the mean. Annotations are colour-coded according to cognitive domains, as indicated in the legend. The videogame annotations are shown in orange. (C–F) show within-participant correlations obtained from session-level maps built using a varying number of runs. They overall show diminishing returns, with the addition of the 5th run improving consistency less than the addition of a 2nd run.

We assessed the robustness of first-level GLM beta maps generated for the same videogame annotations across individuals and recording sessions. To provide a meaningful reference, we also analyzed first-level beta maps derived from more conventional annotations from a battery of localizer tasks in the *hcptrt* dataset. Pearson correlations were computed between pairs of session-wise and run-wise beta maps for the same annotation, considering both videogame (*shinobi*) and *hcptrt* events (Fig. 7A, B). A dataset of the correlations are available in Supplementary Materials (SM4).

Distributions of within-participant correlations for the 28 annotations analyzed varied widely, ranging from r = .666 (SD = .119) for the *hcptrt* relational task to r = .0140 (SD = .188) for inter-annotation comparisons, which served as a lower-bound baseline. Among the 7 game-event annotations, three ranked within the top 10 highest average correlation values: *Kill* (r = .500; SD = .196), *HIT* (r = .495; SD = .186), and *DOWN* (r = .470; SD = .192). The remaining four game-events annotations achieved moderate positive correlations: *RIGHT* (r = .389; SD = .173), *JUMP* (r = .372; SD = .175), *LEFT* (r = .251; SD = .182), and *HealthLoss* (r = .215; SD = .186). The four low-level regressors also showed moderate-to-strong within-participant consistency: *Button press* ranked third overall (r = .509; SD = .175), followed by *Audio envelope* (r = .472; SD = .176), *Luminance* (r = .438; SD = .188), and *Optical flow* (r = .361; SD = .179). *Inter-annotation* comparisons yielded low correlations (r = .140; SD = .188), confirming the distinctiveness of annotation-specific neural patterns. A detailed breakdown of within-participant correlations across all pairs of maps is available in SM3 Supplementary Figure S3.

Between-participant correlations were generally lower than within-participant correlations, spanning from r = .385 (SD = .094) for the relational task to r = .067 (SD = .057) for *LEFT*. Among the game-event annotations, *Kill* (r = .196; SD = .095), *HIT* (r = .190; SD = .096), and *DOWN* (r = .186; SD = .096) showed the highest between-participant consistency, followed by *JUMP* (r = .148; SD = .065) and *RIGHT* (r = .137; SD = .075). *HealthLoss* (r = .068; SD = .056) and *LEFT* (r = .067; SD = .057) showed weak or negligible between-participant correlations. The low-level regressors yielded between-participant correlations broadly comparable to those of the game-event annotations: *Button press* (r = .218; SD = .096), *Audio envelope* (r = .209; SD = .087), *Luminance* (r = .200; SD = .095), and *Optical flow* (r = .108; SD = .068). *Inter-annotation* comparisons resulted in near-zero correlation (r = .074; SD = .097).

Figure 7C–F show within-participant correlations for each of the 11 annotations as a function of the number of runs aggregated into each session-level map (from 1 to 5 runs), separately for each participant. Across almost all annotations and participants, within-participant correlations increased monotonically with the number of runs, with the largest gain occurring between 1 and 2 runs. Subsequent additions yielded progressively smaller improvements, consistent with a pattern of diminishing returns: the benefit of a fifth run was substantially smaller than that of a second (and even decreased consistency for sub-01 and for 2 annotations of sub-06). This pattern was consistent across participants and across both game-event and low-level feature annotations, though the asymptotic level varied: annotations with higher overall reliability (e.g., *Kill*, *HIT*) reached higher plateaus than those with lower reliability (e.g., *HealthLoss*, *LEFT*). These results suggest that a minimum of 2–3 runs per session is sufficient to obtain stable within-participant brain maps for videogame annotations, with limited additional benefit beyond that.

These results indicate that within-participant consistency of brain maps derived from videogame events is comparable to that of established functional localizer tasks, though they are not necessarily the highest overall. The lower between-participant correlations highlight the personalized nature of neural representations elicited by videogame interactions. The overall findings suggest that videogame-derived brain maps exhibit reliability at the individual level while preserving event-specific distinctiveness.

### 3.5 Game events activation maps are discriminable

**Fig. 8.**
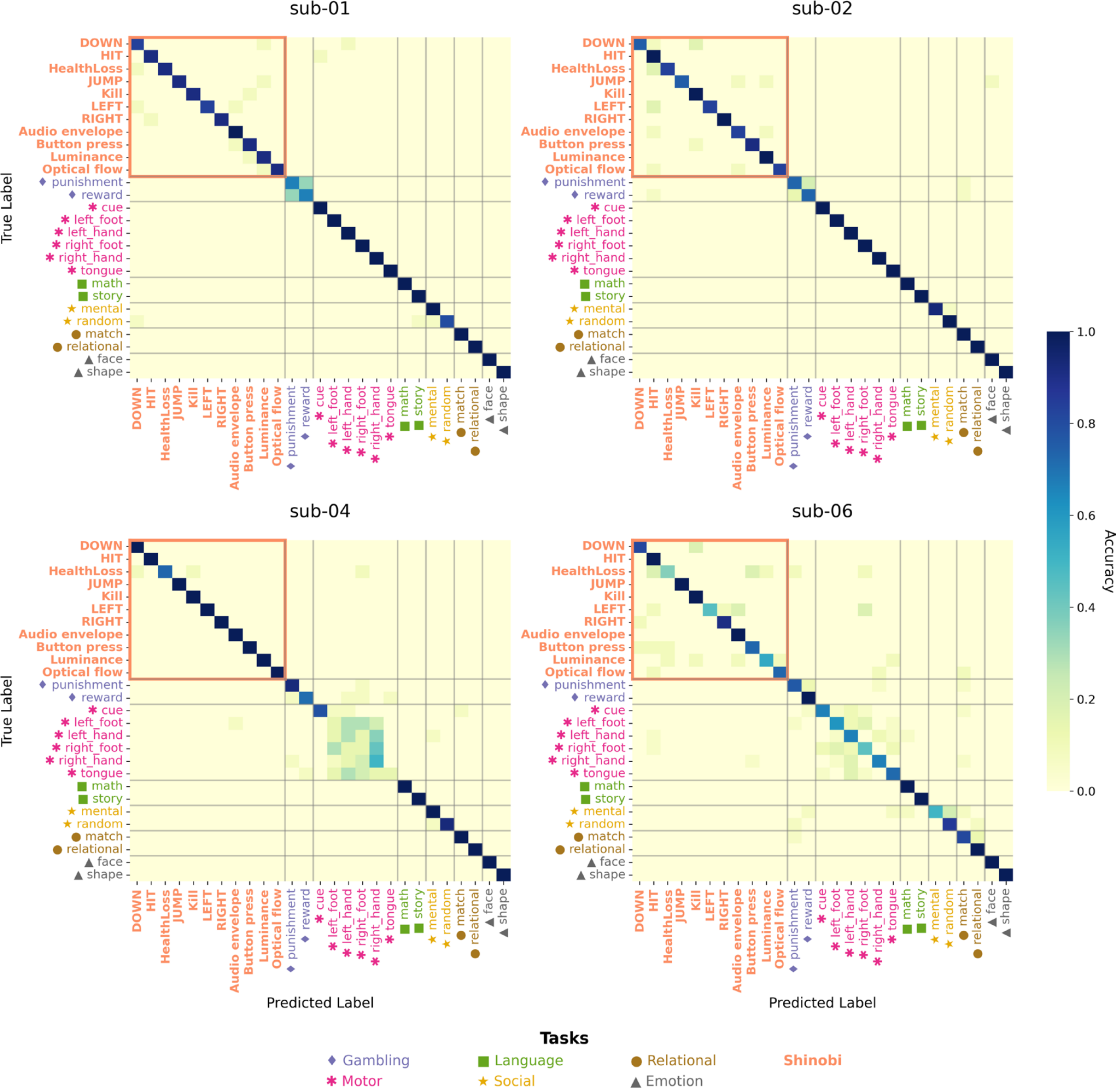
Confusion matrices of MVPA classifications. A linear SVM was trained to classify Z maps from session-wise game annotations and run-wise HCP test–retest annotations, and the models were evaluated with a Leave-One-Group-Out cross-validation. For all game annotations, the classifier correctly identified the true classes above chance level, with occasional confusions between game-related and motor-related maps.

We used a linear support vector machine (SVM) classifier to test whether fMRI activation maps could support reliable classification between different game events. The classification was conducted separately for each participant using a leave-one-session-out cross-validation approach. Specifically, an SVM multiclass model was trained on session-wise Z maps from Shinobi gameplay and tested on run-wise activation maps from the HCP test–retest dataset. Classification performance was evaluated based on the most frequently predicted class across folds for each annotation and participant (Fig. 8). The trained classifiers and per-fold confusion matrices are available in Supplementary Materials (SM5).

Overall, activation maps exhibited high discriminability across annotation types (all p < 0.001). *Kill* and *HIT* were classified with the highest accuracy among game events (mean accuracy = 0.98 across participants), followed by *RIGHT* (0.96) and *JUMP* (0.92). *DOWN* (0.85), *LEFT* (0.78), and *HealthLoss* (0.71) showed comparatively lower accuracy, with the largest drops observed in sub-06 (*HealthLoss*: 0.36, *LEFT*: 0.46) and sub-02 (*DOWN* and *JUMP*: 0.75). The four low-level regressors were also reliably classified, with *audio envelope* achieving the highest accuracy (0.96), followed by *button press count* (0.89), *optical flow* (0.87), and *luminance* (0.87). In most cases, misclassifications of game events involved other game events. Other classification errors on game events were predictions of the motor task, suggesting partial representational overlap with the motor domain. These results confirm that automatically annotated game events elicit distinctive, task-specific activation patterns that can be robustly decoded from fMRI data.

## 4 Discussion

Four participants played three levels of *Shinobi III: Return of the Ninja Master* (1993) during fMRI scanning. Gameplay was implemented using a console emulator integrated with the gym-retro library (Nichol et al., 2018) and controlled via an MRI-compatible gamepad (Harel et al., 2023). BOLD activity was modeled using automated annotations derived directly from the game’s RAM variables. As a proof-of-concept, we generated interpretable, consistent, and distinct brain activation maps linked to these automatically extracted game events and player actions, demonstrating the feasibility and scalability of this novel annotation approach.

### 4.1 Automated content annotation during naturalistic videogame play

By mining the game’s internal memory states, we automatically annotated both player actions and game events. Events such as defeating an enemy (*Kill*) and losing health (*HealthLoss*) were recorded alongside player actions, including character movement, jumping (*JUMP*), and hitting (*HIT*). The *Kill* and *HealthLoss* events represent positively and negatively valenced occurrences, respectively, analogous to manually annotated events in previous studies (Cole et al., 2012; Kätsyri et al., 2013a, 2013b; Zhang et al., 2021). Unlike these earlier efforts, our annotations were generated automatically from an unmodified commercial videogame, enabling seamless scalability to a substantial dataset (32 h of cumulative gameplay), from which we extracted thousands of event instances per participant.

This automatic annotation strategy represents a key departure from previous studies that relied on custom-built games or manual annotation pipelines. For instance, Cole et al. (2012) analyzed brain responses during gameplay in *Re-Mission*, a serious game designed for cancer patients, collecting 6–7 h of fMRI data across 57 participants. Kätsyri et al. (2013a, 2013b) manually annotated around 15–20 events per condition from 11 participants playing *BZ-Flag*. In contrast, our method operates on an unmodified commercial game, scales seamlessly across 32 h of data, and yields thousands of precisely timed (frame-level) event annotations per participant, which is orders of magnitude higher than earlier work. Unlike manual annotations, which often involve downsampling and introduce timing imprecision, our automated approach retains high temporal fidelity, making it suitable not only for fMRI but also for high-temporal-resolution modalities like MEG, EEG, or iEEG.

That scaling factor enabled our analysis to yield interpretable and sensitive maps even for rare events like *HealthLoss*. This suggests that automated annotation not only supports scalability, but can retain the sensitivity of more labor-intensive methods. Nonetheless, imbalances in event prevalence should be handled with care when making contrasts, especially given the heightened salience and stronger neural responses associated with rare, unexpected events (Bueti et al., 2010; Matthews & Gheorghiu, 2016).

### 4.2 Player actions and game-events induce widespread activations including visual and motor cortices

Our GLM analyses revealed a distributed pattern of visual and sensorimotor activations, most consistently involving occipital regions (e.g., cuneus, lingual gyrus) and parietal cortices (e.g., precentral and postcentral gyri). This finding is consistent with the visuomotor demands of playing an action game like *Shinobi III: Return of the Ninja Master*, and mirrors cortical recruitment patterns previously documented in the literature (e.g., Li et al., 2016). Videogame players are known to exhibit enhanced visuomotor skills compared to non-players, along with structural and functional differences in relevant brain regions (Choi et al., 2020). Moreover, videogame training has been shown to induce changes in brain structure and function within the cognitive domains engaged by gameplay (Brilliant et al., 2019).

Player actions such as *HIT* and *JUMP* were associated with activation in the supplementary motor area and cerebellum. These regions are central to the human motor system and play crucial roles in motor preparation, voluntary control, visuomotor adaptation, and temporal prediction (Nachev et al., 2008; Popa & Ebner, 2019; Tanaka et al., 2021; Tzvi et al., 2022). In particular, the observed activation of the supplementary motor area during player-initiated actions supports prior evidence that this region is engaged when motor commands are self-generated rather than externally driven (Nachev et al., 2008; Orban et al., 2015). The *HIT*, *JUMP*, and *Kill* events also elicited activation in the superior frontal gyrus, a region implicated in the generation of complex motor sequences (Martino et al., 2011). Zhang et al. (2021) reported that kill events in a first-person shooter elicited distributed activation patterns, including the supplementary motor area and frontal eye fields—regions associated with inhibitory control and oculomotor processing (Diarra et al., 2019).

Kätsyri et al. (2013a) found that success (kills) and failure (deaths) events modulated activity in ventromedial and orbitomedial regions of the prefrontal cortex, areas implicated in reward processing (Platt & Padoa-Schioppa, 2009) potentially reflecting affective and attentional responses to task feedback. In our dataset, one participant exhibited anterior cingulate activation in response to health loss, potentially reflecting the processing of negative emotional valence (Rolls, 2019). To directly probe this overlap, we compared *Kill*-related activations against a canonical reward condition from the *hcptrt* gambling task (Fig. 6C). *reward* yielded substantially more widespread cortical activations than *Kill*, likely reflecting the considerably higher statistical power afforded by the *hcptrt* design’s rather than a fundamentally broader neural response. *Kill*-unique activations were spatially more focal, concentrated in sensorimotor and posterior visual regions. Combined with the *Kill* vs. *audio envelope* comparison (Fig. 6B), which showed that much of the *Kill* response is shared with low-level auditory features, these findings suggest that *Kill*-related activations represent a more specific signal embedded within a broader landscape of stimulus-driven and general task-related responses.

Most annotations also consistently engaged the precuneus and middle temporal gyrus, two regions not typically highlighted in primary sensorimotor or visual processing. The precuneus is a functionally heterogeneous structure whose subdivisions span sensorimotor, cognitive/associative, and visual networks (Margulies et al., 2009; Zhang et al., 2023), while the middle temporal gyrus is a recognized hub for audiovisual and action-feedback integration (Scheliga et al., 2022; van Kemenade et al., 2019). Their shared recruitment across both player actions and game outcomes likely reflects the continuous multimodal integration demands of gameplay, and illustrates how a single naturalistic task can simultaneously engage the full processing chain from early sensory encoding to higher-order integration.

Collectively, these results converge with existing literature, showing that brain activity patterns identified via automated annotation correspond to previously reported neural correlates of gameplay. This provides strong evidence that automated annotations can validly and effectively model relevant neural processes.

### 4.3 Event-related videogame activations show test-retest consistency, and are participant-specific

Our analyses revealed that brain activation patterns derived from automatically annotated game events were not only consistent across sessions, but also carried clear participant-specific signatures. This level of test-retest reliability, demonstrated using entire sessions as independent folds, is made possible by the unprecedented scale of the CNeuroMod dataset. Such large individual datasets allow us to examine both the reproducibility and individuality of activation patterns in a way that is rarely feasible in fMRI studies. While metrics such as ICC would provide a more formal reliability framework, they require a sufficient sample size to robustly estimate between-participant variance. Our design, with few participants but many sessions per participant, is better suited to multivariate spatial correlation approaches that assess the consistency of whole-brain patterns within and between individuals.

We observed that while certain annotations (e.g., *HIT*, *Kill*) led to highly consistent activation patterns across sessions, the strongest signal came from intra-individual similarity: each participant showed a reliable “neural fingerprint” across time. This participant specificity suggests that the neural encoding of complex game events is both stable and individualised. Low-level regressors (*button press count*, *audio envelope*, *luminance*) also exhibited within-participant consistency comparable to or exceeding that of several *hcptrt* localizer tasks, suggesting that part of the reliability observed for game-event annotations may be driven by their correlation with stable low-level stimulus features.

Notably, this individualization likely reflects more than just biological variability. Because videogames are interactive and open-ended, players shape their own experience through strategy, pacing, and response to in-game challenges, effectively constructing personalized versions of the task. These player-specific gameplay trajectories can drive idiosyncratic cognitive demands, contributing to the observed neural distinctiveness. In this context, videogame-based paradigms offer a powerful lens for investigating how specific brains engage with dynamic, ecologically valid environments, with promising applications in the study of individual differences and clinical populations.

Between-participant conjunction maps (SM3 Supplementary Fig. S2) nonetheless reveal that several activation patterns, particularly in visual and sensorimotor cortices, were consistently observed across all four participants for most annotations. This suggests shared neural substrates despite the overall dominance of individual-specific signatures. However, our design with only four participants does not allow for a formal statistical assessment of group-level effects beyond these descriptive observations.

An additional analysis examining how within-participant map consistency scales with the number of runs per session revealed a pattern of diminishing returns: the largest gain in reliability occurred when moving from a single run to two runs, with each subsequent run contributing progressively less. It also shows that even with a few runs reliable brain maps can be derived, depending on the type of annotation. This finding has practical implications for experimental design: the frequency and regularity of the events of interest should be carefully considered when determining the volume of data required to achieve stable, reliable brain maps for a given annotation.

### 4.4 Event-related videogame activations are annotation-specific

The MVPA classification of session-wise activation maps revealed that neural patterns linked to distinct game annotations were highly discriminable. This is especially striking given that all annotations originated from a single, continuous task rather than from separate experimental conditions. Comparable classification accuracy has previously been achieved across very different tasks (e.g., motor vs language) in large datasets like the Human Connectome Project (Porter et al., 2023; Rastegarnia et al., 2023). Here, we show that similarly distinct neural signatures can be recovered from different aspects of a single task, further highlighting the richness of cognitive states evoked during gameplay.

This discriminability is noteworthy given the substantial temporal co-occurrence of several game events (e.g., *DOWN*, *HIT*, and *Kill*; r up to 0.81). Although collinearity was handled by fitting each annotation in a separate GLM, the resulting whole-brain patterns remained highly distinguishable, consistent with the known sensitivity of multivariate decoding to distributed, condition-specific signals that univariate approaches cannot isolate (Haxby et al., 2001; Jimura & Poldrack, 2012). The four low-level regressors were also reliably classified, with accuracies comparable to several game-event annotations, confirming that these continuous sensory and motor features engage spatially distinct brain patterns rather than capturing undifferentiated nuisance variance.

The lower classification accuracy for *LEFT* and *HealthLoss*, particularly in sub-06, likely reflects insufficient data: both events were infrequent, and the reliability scaling analysis (Fig. 7C–F) showed that annotations with fewer occurrences reach lower reliability and do not plateau in our dataset. For *HealthLoss* specifically, additional variability may arise from the context-dependent nature of outcome processing (Fouragnan et al., 2018), though disentangling data-volume and encoding-variability contributions would require denser event sampling.

These results position automated videogame annotation as a viable approach for cognitive state decoding within a single continuous naturalistic task, without the artificiality of blocked designs. The robust within-participant patterns observed here, combined with evidence that naturalistic paradigms preserve or enhance individual neural signatures (Finn, 2020; Vanderwal et al., 2017), suggest promising applications in single-participant brain mapping and the study of individual differences.

### 4.5 Limitations

Our approach relies on reverse engineering memory addresses to identify the location of relevant game variables. Although this mapping can be exhaustive in principle, it is often incomplete in practice, spanning only a limited subset of variables. Depending on the game software’s architecture and physical constraints of the support (size, bit-depth), certain variables can be encoded in unintuitive ways and be tedious to recover. This process however only has to be performed once, and partial or complete variables mapping can sometimes be available online through resources from the modding community, limiting the amount of work required to access experimentally meaningful variables.

Memory-based annotations provide a temporal resolution equivalent to the games framerate, which was 60 Hz in our case. Although this temporal resolution is far beyond that of fMRI, other brain measurement techniques such as MEG and EEG offer millisecond resolution. Using our annotations framework in these cases requires precise logging of individual frames’ timing, which enables matching a variable change to the exact moment at which the corresponding frame is displayed. Given the continuous nature of gaming stimuli however, events commonly spans multiple frames, and stimulus onset as perceived by the player (e.g., feedback on a given action, appearance of an enemy on screen) is not always perfectly aligned with the frame corresponding to the variable change and can vary depending on animations and inherent delays.

The GLM approach used in the current set of analyses carries inherent limitations that constrain its application to more complex event structures. Because GLMs assume low collinearity among regressors, we had to analyse each annotation separately, an approach that may not scale well as annotation granularity increases. In practice, some collinearity is unavoidable: in dynamic tasks like gameplay, events unfold in a causal sequence and are not temporally isolated. GLMs impose a linear relationship between regressors and BOLD signals, which may not adequately capture the nonlinear dynamics of brain responses during naturalistic, context-dependent events. Prior studies have demonstrated such nonlinearities in complex motor tasks (Alahmadi et al., 2016) and in contexts where event meaning varies (Orban et al., 2015; Tsivilis et al., 2003). Exploring nonlinear modeling, such as brain encoding with AI models (Ahmadi et al., 2024; Cross et al., 2021; Kemtur et al., 2023), may offer deeper insights into how diverse cognitive processes interact during gameplay (Gonzalez-Castillo et al., 2012). Additionally, model-free approaches such as finite impulse response (FIR) models could be used to characterize the shape of event-locked BOLD responses without assuming a canonical hemodynamic profile, providing a complementary validation of the activations identified through our GLM analyses.

### 4.6 Relevance and future directions

The present analyses focused on low-level, discrete game events that map basic perceptual and motor components of gameplay. However, videogame play notoriously engages higher-order processes, including learning, reasoning, planning, and affective evaluation, that our current annotations do not capture (Schrodt et al., 2017). Extending this framework to study learning, for instance, would require segmenting platformer levels into smaller gameplay units based on recurring design elements (e.g., platform layout, enemy types; Smith et al., 2008), chunking level repetitions, and tracking progress along specific performance metrics; we explore this direction in a companion benchmark proposal (Harel et al., 2025). Affective processing represents another natural extension: the *Kill* and *HealthLoss* annotations already serve as proxies for positively and negatively valenced outcomes, and physiological data (cardiac, respiratory) were recorded concurrently with fMRI in the present dataset. Other datasets acquired within the CNeuroMod project further include phenomenological questionnaires alongside physiological recordings, and will be the subject of future work. Automated annotations from memory can support these efforts at scale, and when combined with the gym-retro library, which enables programmatic control over the emulator’s state, a game can be “taskified” (i.e., configured into a well-controlled experimental task; Allen et al., 2024) to target specific aspects of higher-order cognition.

A notable strength of videogame paradigms is their capacity to generate large volumes of repeated events under sustained participant engagement. In the present dataset, a single annotation such as Kill was observed approximately 400 times per session and over 4,000 times per participant across the full experiment. For comparison, large-scale visual neuroscience datasets typically present on the order of thousands of unique images with only a handful of repetitions each: the Natural Scenes Dataset presents ~10,000 images shown 3 times each (~30,000 total trials; Allen et al., 2022), CNeuroMod-THINGS presents ~4,300 images shown 3 times (~13,000 trials; St-Laurent et al., 2026), and THINGS-data presents ~1,854 images shown once (Hebart et al., 2023). A single videogame event type thus yields a comparable number of observations to the entire stimulus set of these datasets, but with each instance occurring in a unique gameplay context involving different enemies, spatial configurations, and action sequences. This contextual variability, combined with intrinsic participant motivation, makes videogames well suited for studying how the brain generalises representations across naturalistic conditions. Moreover, because gameplay simultaneously engages perception, decision-making, motor planning, and affective evaluation as a coupled dynamical system, it offers a window into cognitive processes that are typically studied in isolation through separate experimental tasks.

Importantly, videogame research can draw on a robust ecosystem of design practices, community-developed tools, and analytic frameworks, which makes implementation both flexible and scalable. Videogame paradigms are also inherently engaging and accessible, lending themselves well to use in populations for whom conventional cognitive tasks may be unsuitable such as children (Vanderwal et al., 2019) or clinical populations (Chitale et al., 2022; Peñuelas-Calvo et al., 2022). Moreover, games may uncover compensatory mechanisms in individuals with cognitive challenges, offering a promising avenue for therapeutic applications (Han et al., 2009). Because they are embedded in context-sensitive, continuous tasks, videogames also allow for the detection of subtle behavioural and neural shifts that might be missed in more constrained paradigms. Our approach, which enables scalable, automated annotation across thousands of games, opens the door to broader, more inclusive applications of these tools.

Videogames are also well suited to studying states of intense task engagement. Psychological flow, characterized by deep absorption, loss of self-consciousness, and a sense of effortless control, is notoriously difficult to induce and observe under laboratory conditions (Abuhamdeh, 2020), yet arises readily during videogame play (Khoshnoud et al., 2020; 2022). Unlike most experimental tasks, videogames allow researchers to recruit individuals with genuine expertise from the general population, ensuring that the skill levels required for flow to emerge are ecologically represented. Moreover, because videogames are inherently engaging, participants can sustain performance over extended acquisition sessions, increasing the likelihood that rare, spontaneous phenomena such as flow will occur in sufficient quantities to be analysed. Combined with physiological recordings and automated annotations, the fine-grained performance metrics available through game analytics can complement subjective reports to help identify and characterise these episodes, constituting a promising avenue for future studies.

These paradigms also hold significant promise for bridging human and artificial neural systems (Bellec & Boyle, 2019). Artificial neural networks (ANNs) trained on multimodal gameplay data can be used to predict neural activity, as shown by Cross et al. (2021), who demonstrated correspondence between ANN and human brain responses in sensorimotor regions. Yet, the interpretability of ANN models remains a challenge due to the complexity of their internal representations. Automated annotation strategies like ours provide a pathway toward greater interpretability by linking brain responses to specific, meaningful in-game events (e.g., “killing an enemy” or “losing health”). For instance, if an ANN layer becomes selectively responsive to Kill events, we can begin to isolate the visual or cognitive components underlying this prediction. This synergy between data-driven modeling and semantically grounded annotations enhances the explanatory power of brain–ANN alignment.

In addition, videogame tasks can support advanced machine learning approaches such as imitation learning (Hussein et al., 2017), where models learn to replicate human gameplay. These models can be constrained or validated using brain activity, enabling the development of brain-aligned ANNs that mirror individual players’ neural patterns (Freteault et al., 2025; Kemtur et al., 2023). Our approach, with its combination of naturalistic stimuli and rich, structured annotations, offers a promising testbed for training and evaluating such models. Finally, the abundance of annotated data generated by this approach facilitates exploratory analyses and hypothesis generation, helping to uncover the neural mechanisms underlying real-world behaviour in a scalable, interpretable way.

### 4.7 Conclusion

Our results show that automated annotations derived from unmodified retro videogame play can reliably produce interpretable brain activation maps. These maps engage biologically plausible networks that are specific to distinct in-game events, demonstrating that dynamic, naturalistic stimuli like videogames can be leveraged to model reproducible and functionally meaningful neural responses.

Although this study focused on Shinobi III: Return of the Ninja Master, the methodology generalizes readily to the entire gym-retro library, spanning over 1,000 games across 10 different consoles, and can be extended to many other platforms. We hope that this work will encourage broader adoption of commercial videogames in cognitive neuroscience and contribute to a richer understanding of brain function under naturalistic, ecologically valid conditions.

## Supplementary Material

Supplementary Material

## Data Availability

All datasets used in this study are part of the CNeuroMod dataset (https://www.cneuromod.ca/). The code developed for this research is publicly available on GitHub (https://github.com/courtois-neuromod/shinobi_fmri) and archived on Zenodo for long-term preservation (https://doi.org/10.5281/zenodo.15628556). Processed datasets used to generate the tables and figures in this manuscript are provided as Supplementary Material accompanying the paper.

## Appendices

**Appendix Table A1.** Descriptive table of the *shinobi_training* dataset.

		sub-01	sub-02	sub-04	sub-06	Total
level-1	N	164	263	57	34	518
	cleared	101	211	40	17	369
	duration (h:m:s)	07:31:27	12:56:27	03:56:13	02:26:04	26:50:11
level-4	N	108	288	98	54	548
	cleared	39	54	37	5	135
	duration (h:m:s)	03:01:36	10:39:10	05:33:00	02:33:39	21:47:25
level-5	N	116	51	57	36	260
	cleared	86	16	26	9	137
	duration (h:m:s)	05:00:33	02:58:27	03:13:23	01:54:04	13:06:27
Total	sessions	53	110	24	15	202
	N	388	602	212	124	1326
	cleared	226	281	103	31	641
	duration (h:m:s)	15:33:36	26:34:04	12:42:37	06:53:46	61:44:04

### A.1 Performance Comparison of Training vs Scanning Phases

Training sessions were ordered chronologically by timestamp and scan sessions by acquisition order; each sequence was split at its midpoint into a first and second half. Three consecutive half-pairs were compared within training, from training to scanning, and within scanning, using two-sided Welch’s *t*-tests with Benjamini-Hochberg FDR correction applied across all comparisons per participant. This was done separately for each level and metric (final score, proportion cleared, health loss, and screen-clearing rate).

### A.2 Skill Acquisition During Training Transfers to the Scanner

Participants trained at home for 59–84 weeks prior to scanning (16–110 sessions depending on participant), followed by 11–12 MRI scan sessions. All four participants improved substantially across the training phase, with significant gains in proportion of screens cleared and final score at levels 1 and 4 in all participants (all *q* < .05; see Appendix Fig. A1). Level-5 improvements were less consistent, likely due to ceiling effects and fewer training sessions. Skills transferred robustly to the scanner at level 1, with all participants entering the scan phase well above their end-of-training performance. Transfer at level 4 was more variable: two participants showed slightly lower performance at scan entry than at training end, possibly reflecting a brief adjustment to the scanner environment. Performance was largely stable across the scanning period, with 32 of 40 within-scan comparisons non-significant. A small number of continued improvements were observed in a few participants at levels 1 and 4, consistent with the more regular weekly scan schedule providing additional structured practice.
