# Supplementary material for: Gamer in the scanner: Event-related analysis of fMRI activity during retro videogame play guided by automated annotations of game content
Source: Imaging Neurosci (Camb). 2026 Jun 1;4:IMAG.a.1256. doi: 10.1162/IMAG.a.1256 (PMC13228873; doi:10.1162/IMAG.a.1256)
Supplement: Supplementary Material [file IMAG.a.1256_supp.zip › SM3_figuresandtables.pdf]

Supplementary figures and table

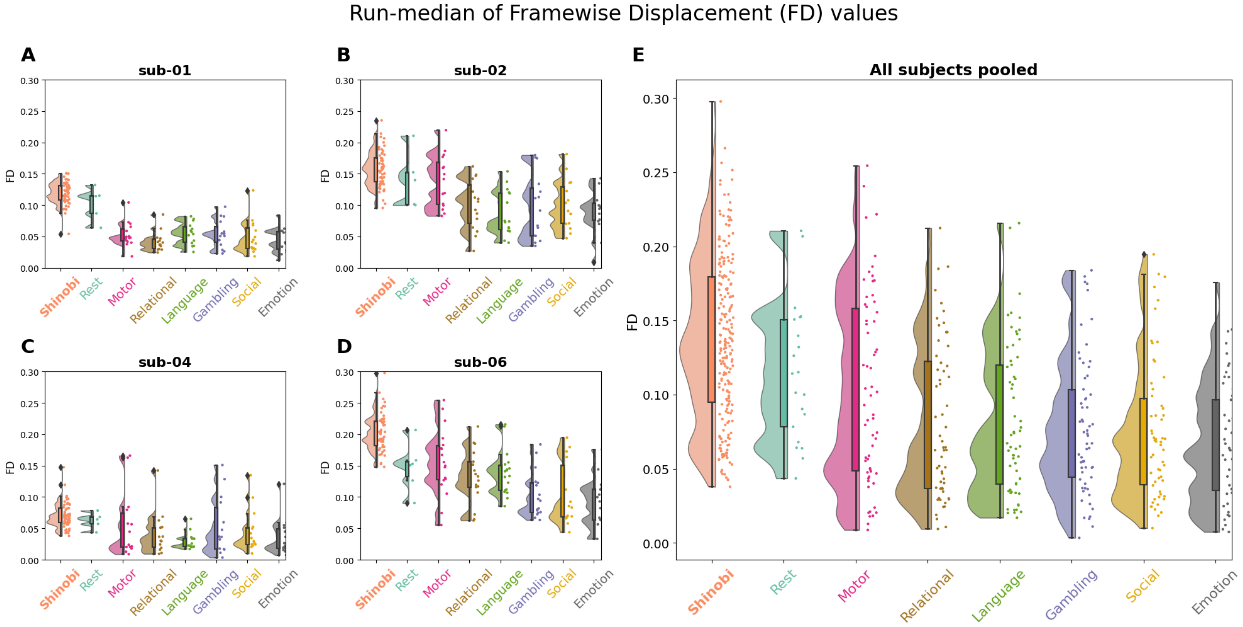

Supplementary Figure 1. Run-median of Framewise Displacement (FD) values, indicative of motion levels in the *shinobi* and *hcptrt* datasets. The values observed in the *shinobi* dataset were comparable to the high end of the *hcptrt* subdatasets, such as the resting state (Rest) and Motor tasks, a pattern that stays consistent across participants.

Label	Annotation (participant count, max. 4)
Occipital_Mid_L	RIGHT (4); HIT (3); optical (3); LEFT (3); DOWN (2); Kill (2); JUMP (2); audio (2); button (2); luminance (2)
Parietal_Sup_L	JUMP (3); optical (3); RIGHT (3); LEFT (2); Kill (2)
Calcarine_L	JUMP (4); RIGHT (3); optical (3); DOWN (2)
Occipital_Sup_L	JUMP (3); LEFT (3); RIGHT (3); optical (3)
Precentral_L	HIT (3); RIGHT (2); luminance (2); optical (2)
Temporal_Mid_R	HIT (3); Kill (3); HealthLoss (2); audio (2)
Calcarine_R	JUMP (2); Kill (2); optical (2)
Frontal_Sup_2_R	DOWN (2); HIT (2); JUMP (2)
Occipital_Mid_R	optical (3); JUMP (2); Kill (2)
Occipital_Sup_R	JUMP (2); RIGHT (2); optical (2)
Parietal_Sup_R	RIGHT (3); optical (3); JUMP (2)
Precuneus_L	DOWN (3); HIT (2); Kill (2)
Precuneus_R	DOWN (3); Kill (3); RIGHT (2)
Temporal_Sup_R	HIT (2); HealthLoss (2); button (2)
Cuneus_L	JUMP (3); LEFT (3)
Cuneus_R	JUMP (2); RIGHT (2)
Lingual_L	RIGHT (2); optical (2)
Postcentral_L	HIT (2); JUMP (2)
Precentral_R	JUMP (3); HIT (2)
SupraMarginal_R	HealthLoss (2); button (2)
Cerebelum_8_R	RIGHT (2)
Cerebelum_9_R	RIGHT (3)
Frontal_Inf_Oper_L	luminance (2)
Frontal_Mid_2_R	DOWN (2)
Frontal_Sup_2_L	RIGHT (2)
Lingual_R	optical (2)
Paracentral_Lobule_L	RIGHT (2)
Supp_Motor_Area_L	RIGHT (2)
SupraMarginal_L	DOWN (2)
Temporal_Mid_L	LEFT (2)

Supplementary Table 1. AAL regions activated across participants and annotations. The table lists brain regions (according to the AAL atlas) that showed

significant activation (z > 3, cluster size > 10) present in at least two participants for a given annotation.

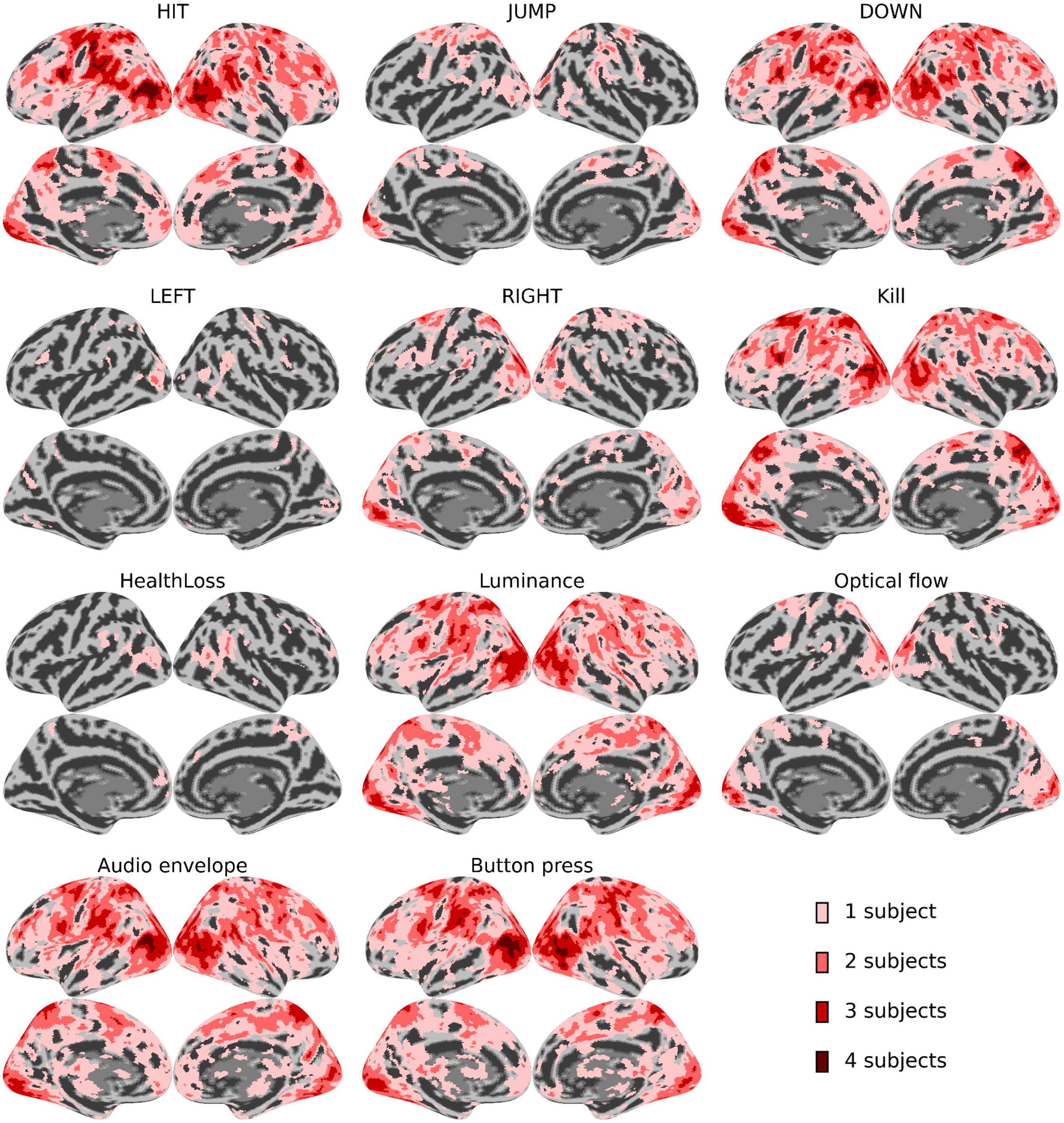

Supplementary Figure 2. Between-participants conjunction maps. These maps show the conjunction across participants of binarized, FDR corrected z-maps obtained for player actions, game events and low-level features. Darker colors indicate voxels where more participants showed significant activations in a given condition.

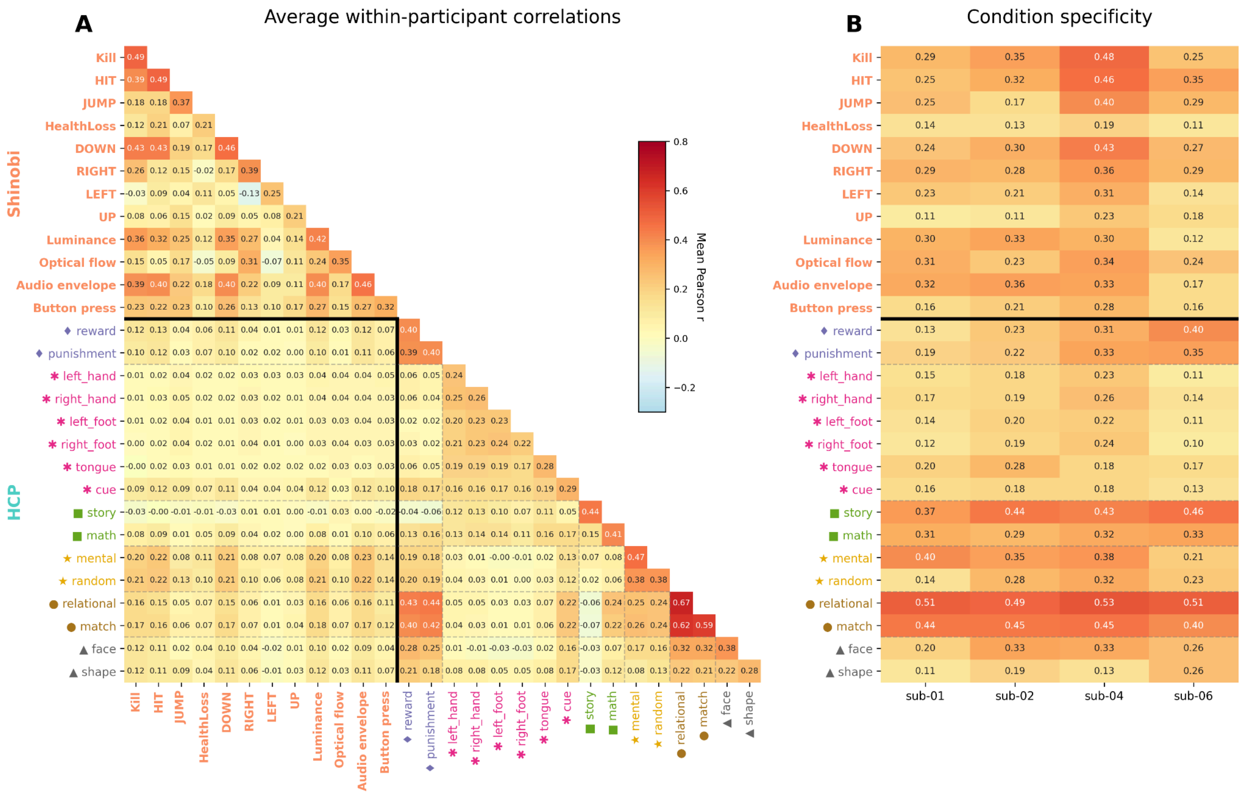

Supplementary Figure 3. Detailed breakdown of within-participants correlations between beta maps across the *shinobi* and *hcptrt* datasets. A Average within-participant correlations for each pair of annotations. **B** Within-participant condition specificity, defined as the difference between within-annotation correlations and between-annotations correlations, computed separately for each participant.
